# Female Presence and Estrous State Influence Mouse Ultrasonic Courtship Vocalizations

**DOI:** 10.1371/journal.pone.0040782

**Published:** 2012-07-18

**Authors:** Jessica L. Hanson, Laura M. Hurley

**Affiliations:** Department of Biology, Indiana University, Bloomington, Indiana, United States of America; Utrecht University, The Netherlands

## Abstract

The laboratory mouse is an emerging model for context-dependent vocal signaling and reception. Mouse ultrasonic vocalizations are robustly produced in social contexts. In adults, male vocalization during courtship has become a model of interest for signal-receiver interactions. These vocalizations can be grouped into syllable types that are consistently produced by different subspecies and strains of mice. Vocalizations are unique to individuals, vary across development, and depend on social housing conditions. The behavioral significance of different syllable types, including the contexts in which different vocalizations are made and the responses listeners have to different types of vocalizations, is not well understood. We examined the effect of female presence and estrous state on male vocalizations by exploring the use of syllable types and the parameters of syllables during courtship. We also explored correlations between vocalizations and other behaviors. These experimental manipulations produced four main findings: 1) vocalizations varied among males, 2) the production of USVs and an increase in the use of a specific syllable type were temporally related to mounting behavior, 3) the frequency (kHz), bandwidth, and duration of syllables produced by males were influenced by the estrous phase of female partners, and 4) syllable types changed when females were removed. These findings show that mouse ultrasonic courtship vocalizations are sensitive to changes in female phase and presence, further demonstrating the context-sensitivity of these calls.

## Introduction

Animal vocal communication is a widespread phenomenon that provides insight into context-dependent signaling and reception [1]–[6]. A recent surge of interest has focused on ultrasonic vocalizations (USVs) of adult laboratory mice as a model for both production of vocalizations and auditory processing [7], [8]. Laboratory mice produce USVs in social contexts, although the extent to which USVs communicate social information is not well resolved [9]–[15]. A more detailed understanding of USVs will allow better investigation of how behavioral context influences both signal production and reception. Furthermore, a greater understanding of how USVs are involved in communication would benefit research on human disorders of vocal communication using genetically modified mice [16]–[18].

The production of vocalizations in mice is broadly context dependent, occurring to the greatest degree during social encounters, including mother-pup interactions and adult intersexual and intrasexual interactions, but also in other contexts such as during physical restraint [10], [12], [14], [15], [19]. In adult social exchanges, USVs are produced at a high rate during male-female interactions. Although both sexes produce USVs, the majority of USVs produced during male-female interactions in the genus *Mus* are believed to be from males [9], [10], [19]–[21]. This was most convincingly demonstrated by a study in which devocalizing males reduced USV production to the level shown by devocalized male-female pairs, and devocalizing females had no effect on USV production [19]. Robust vocalizations by males can be triggered not only by female presence, but also by cues such as female urine [22], [23]. USV production may also correspond to other behaviors exhibited during courtship. For example, the overall number of syllables and even particular syllable types produced by males correspond to particular courtship behaviors such as mounting and social investigation [24], [25]. Females demonstrate interest in USVs by approaching speakers broadcasting recorded USVs and by preferring vocalizing males to silent males [26]–[28]. Females also prefer novel, non-kin USVs over familiar USVs from siblings [23]. All of these findings suggest that USVs have an important role in courtship, potentially facilitating mate attraction or mate choice. Whether the finer structure of courtship vocalizations is important to this role, particularly how spectrotemporal features of syllables correspond to behaviorally important aspects of courtship, is not well understood.

Spectrographic analysis of USVs has shown that mouse vocalizations are highly complex and span a range of spectrotemporal parameters. Syllables grouped by these parameters occur in non-random orders [29]. Furthermore, individuals produce unique vocalizations based on characteristics such as frequency, and these vary over the course of development [28]–[30]. Such observations have been used to propose that the spectrotemporal structure of mouse USVs communicate information important to social interactions [28]–[30]. Using spectrographs to analyze USVs provides an opportunity to assess the functional and behavioral significance of mouse vocalizations at a deeper level. In other model systems of vocal courtship, changes in the frequency, duration, or complexity of vocal signals are important for the signal-receiver interaction [31]–[35]. Here, we tested whether the usage or the spectrotemporal parameters of different syllable types correspond to social information of potential significance to male mice.

We first analyzed correlations between syllable types and other behaviors that occur during courtship, making the prediction that courtship behaviors such as ano-genital sniffing and mounting would be positively correlated with the rate of production of USVs as well as the use of specific syllables. Social information potentially relevant to the courting male was also manipulated in two ways. One of these was to provide males with female companions in different estrous phases. Since male courtship behaviors including the rate of USV production vary across the estrous cycle of females, we hypothesized that differences in the characteristics of USVs might also co-occur across the estrous states [36]–[38]. In addition to using females varying in estrous phase, we removed females from males during courtship sequences, and measured USVs in the female-present and female-absent “sub-contexts.” We reasoned that female presence is a highly relevant cue that may vary over the course of courtship interactions in natural settings [39]. Since courtship USVs show a high level of sensitivity to cues indicating female proximity, we predicted that USVs would change rapidly in response to female removal [22], [23].

## Methods

### Animals

All procedures were approved by the Bloomington Institutional Animal Care and Use Committee (Indiana University, protocol 09-038). CBA/J mice (Jackson Laboratories) aged 2–7 months were used. Nine vasectomized males were studied; 14 females were used as stimulus mice. All mice were housed individually on a 14∶10 hour light:dark cycle. Food was provided *ad libitum*. All animals participated in 5–10 male-female interactions prior to experimentation, and only animals displaying mounting behavior (males) or allowing mounting behavior (females) were subsequently used in this study. Males and females both interacted with multiple partners throughout the pre-experiment training process, and specific male-female pairings were repeated multiple times. Only pairs of animals that had interacted at least once during pre-experiment interactions were paired during experiments. Thus, all experimental interactions were between familiar mice.

Home cages containing male mice were placed in a larger recording chamber for at least one half hour prior to behavioral and acoustic recording to allow males to acclimate to the testing room. Each male was monitored (audio and video recording) for 5 minutes prior to a female being added to the cage. The male-female interaction took place over 5 minutes, after which the female was removed. The male mouse was then monitored for an additional 5 minutes. On experimental days, both males and females were recorded up to 3 times, but never the same pair on the same day. The order of male-female interactions were arranged to avoid having the same female be the first female each male interacted with on a particular day or across experimental days. Each animal was allowed to rest for at least one hour alone in its home cage between trials on the same day.

Females were assessed for estrous phase on the day of experimentation by vaginal lavage. Smears were stained with Giemsa and analyzed under a microscope. Phases were determined by the relative number of cell types visible. Presence of leukocytes indicated diestrus, presence of only cornified epithelial cells indicated estrus, and presence of both cornified and nucleated epithelial cells indicated proestrus [40]. Mice were analyzed for estrous phase every day, even non-experimental days, because most did not follow a regular 4 day cycle. Because estrous state could not be predicted before experimental days, states were represented randomly in the experimental design, without experimenter bias. Daily staging also allowed for standardized handling across individuals in the study. Data from females in the phases of proestrus, estrus, and diestrus were compared in this study. Diestrus rodent females are not considered sexually receptive, whereas females have been deemed behaviorally receptive in proestrus and estrus, although expression of receptivity is complex and variable [36], [41].

### Sound/video Recording

The video camera and microphone were positioned above the recording cage. Mouse vocalizations were recorded with a condenser microphone (CM16/CMPA, Avisoft Bioacoustics) and sound card (250 kHz sample rate, UltraSoundGate 116 Hb, Avisoft Bioacustics). Video was recorded with a CCD video camera (30 fps), Q-see 4 channel DVR PCI video capture card, and SuperDVR software (Q-See, Digital Peripheral Solutions Inc.). Behaviors were analyzed from video clips at a later time by an observer blind to mouse identity and female state using ODLog software (Macropod Software).

### Behavior

Behaviors of both male and female mice were measured during the 5 minute interaction, while only the male was measured in the 5 minutes after interaction. Since in most cases no syllables were produced before interaction, this segment of time was not analyzed. Nonsocial male behaviors including locomotion, rearing, digging, and USVs were measured both during interaction and after (Table 1). Additional male behaviors measured during the interaction were investigation of the female ano-genital region and mounting. Female behaviors measured were investigation of the male ano-genital region and rejection of the male (kicking or darting away). The amount of time that the male and female spent face to face was also scored; this occurred most often due to the male approaching the female. USVs were also analyzed (see next section).

**Table 1:** Behaviors recorded during and after male-female interactions analyzed by video recording.

Behavior	Description	Context
Locomotion	Male mouse ambulates, displacing himself in space	During and after interaction
Digging	Male mouse visibly moves bedding with forelegs	During and after interaction
USVs	Ultrasonic syllables visible on spectrograph	During and after interaction
Rearing	Male mouse lifts forelegs from ground, either into the air or against the cage wall	During and after interaction
Male investigation of female ano-genital region	The male’s nose is in close proximity to the female’s rear	During interaction
Female investigation of male ano-genital region	The female’s nose is in close proximity to the male’s rear	During interaction
Male and female nose-to-nose investigation	The male and female’s noses are in close proximity to each other, usually as aresult of male advances	During interaction
Female rejection	The female kicks, jerks, or rapidly bolts when in close proximity to the male	During interaction
Mounting	The male places his forelegs on the female’s back, oriented from the rear, whilemaking pelvic thrusts	During interaction

### Ultrasonic Vocalizations

**Figure 1:**
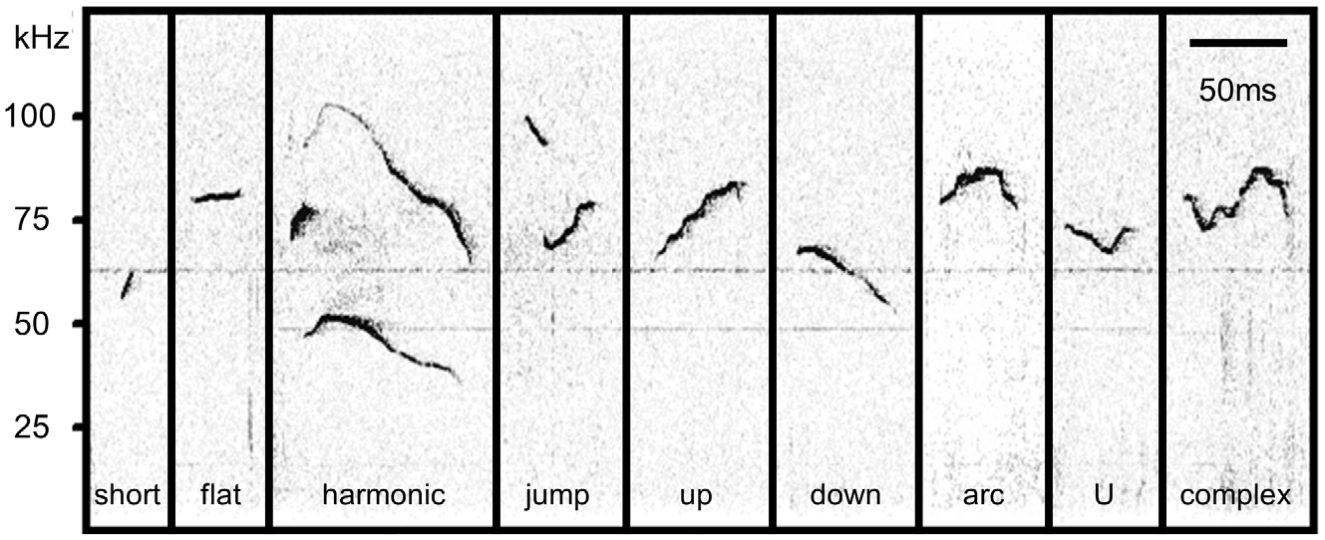
Representative examples of different syllable types. USV syllables were categorized into 9 syllable types based on spectrograph parameters.

USVs were categorized into syllable types using sound spectrographs (Avisoft Bioacustics SasLab Pro software). Spectrographs were generated with an FFT length of 512 and a Hamming style window with 50% overlap. Syllables were sorted into 9 types based on length, bandwidth, and overall shape, adapted from previously described methods [17], [29] (Fig. 1).

**Short** syllables were less than 10 ms in duration.
**Flat** syllables had less than 5 kHz of modulation.
**Harmonic** syllables contained at least one segment with at least one harmonic (most of these also had breaks in frequency).
**Jump** syllables contained at least one break in frequency with no break in intensity (and no harmonics).
**Up** syllables increased in frequency (sweep>5 kHz).
**Down** syllables decreased in frequency (sweep>5 kHz).
**Arc** syllables increased and then decreased in frequency, with the highest frequency reaching >5 kHz above the beginning and end frequencies.
**U** syllables decreased and then increased in frequency, with the lowest frequency reaching >5 kHz below the beginning and end frequencies.
**Complex** syllables contained two or more directional changes in frequency and >5 kHz modulation of frequency.

The following are differences between our procedure and two other studies that used similar categories. The required modulation of 5 kHz for our “complex,” “up,” “down,” “arc (chevron),” and “U (reverse chevron)” syllables is less than the 6 kHz and 6.25 kHz in the other studies, which distributed more syllables from the “flat” category into other categories in our study. The 5 kHz cut-off for our “flat” syllables was intermediate between the 3 kHz and 6 kHz that were used in the other studies. We did not observe syllables matching the descriptions of “noisy syllables” from Grimsley *et al*. and “harmonics,” from Scattoni *et al*. Instead, in our harmonic category we included “composite” and “frequency step” syllables, so long as a harmonic was present. We did observe low frequency harmonic syllables (audible calls); however, we did not include analysis of these syllable types because they appeared to be produced exclusively by females [21].

Duration, bandwidth, and multiple other frequency parameters of each USV syllable were measured. Frequency was measured at the beginning and end of each syllable. The maximum and minimum frequencies of each syllable were also recorded, as well as the dominant frequency (frequency of maximum intensity). For each 5 minute sub-context segment recorded (5 minutes during and 5 minutes after each interaction), the number of syllables of each type was normalized to total number of syllables produced in order to obtain a measure of proportional syllable usage. Analysis of USVs was performed blind to female estrous state.

### Statistics

USV parameters were not normally distributed, so only non-parametric tests were used. The number of USVs produced across trials was highly variable, so percent use of syllable types was calculated instead of using absolute counts. USVs were analyzed for differences among males (Kruskal-Wallis). Each male was measured in 4–6 interactions with different females. The average percent syllable use for each male was compared between sub-contexts (Wilcoxon signed ranks test). Syllable parameters (frequency, duration, and bandwidth) were also compared across males and between sub-contexts (Kruskal-Wallis and Wilcoxon signed ranks test). Although 5 frequency parameters were measured, they were highly correlated (PCA, Table 2), so only dominant frequency was used in our analyses. The relationships between vocalizations and other behaviors were assessed by comparing the average duration of behaviors across 5 minute interactions per male to the average number of syllables of each type within the same 5 minutes (Spearman’s rank correlation). The differences in duration, frequency, and bandwidth during and after interaction were assessed with respect to individual male and female identity to test whether identity could account for variation in change in parameters (Kruskal-Wallis). Wilcoxon signed ranks tests were used to compare the number of syllables occurring in the 10 second time bins before and following mounting. The type and parameters of syllables uttered by males during interactions with females of different estrous phases were compared (Kruskal-Wallis). For each test assessing multiple comparisons, False discovery rate (FDR) post-hoc corrections were made [42]. Since multiple recordings per animal were made on a single day, the number of USVs and percent use of syllable types with respect to order of experiment were assessed, and there was no effect of experiment order on USVs (Kruskal-Wallis, all p-values >0.05 significance level). Although data from a range of ages were collected, correlations between age and syllable use or parameters were not significant, so the data were pooled (all p-values >0.05 significance level; see Table S1 for exact values).

**Table 2:** Principle component analysis on frequency parameters.

Frequency parameter	Weight in component 1 (63%of variance)
Frequency at start	0.818
Frequency at end	0.885
Dominant frequency	0.723
Minimum frequency	0.695
Maximum frequency	0.846

## Results

### Characterization of Adult Male CBA/J Mouse USVs and Individual Variation in Behavior

In order to characterize the vocal repertoire of male CBA/J mice in a courtship context, the USVs of mice were measured during and after male-female interactions. A total of 21964 syllables from 9 CBA/J male mice were grouped into categories based on spectrograph-derived parameters. The most common syllable produced was “up,” which represented 23.81 percent of all syllables (Table 3). A large subset of 21528 syllables was analyzed for parameters of frequency, duration, and bandwidth. USVs ranged from 26.300 to 124.000 kHz, with a mean dominant frequency of 74.662±0.055 kHz. Different syllable types had different dominant frequencies, durations, and bandwidths (Kruskal-Wallis p<0.001; Table 4). The variation in duration across syllable types was large, with “short” syllables averaging 5.8 ms, and “harmonic” syllables averaging the longest at 51.3 ms. In bandwidth, syllables with frequency jumps and harmonics had wider bandwidths than all other syllables (25.2 kHz, 46.9 kHz), but the harmonic category had by far the largest average bandwidth. Thus, the harmonic type syllables were longer, wider bandwidth vocalizations than any other category of syllable (Fig. 1).

**Table 3:** Overall number of syllables per type and percent use of syllables per 10 minute trial.

Syllable type	short	flat	jump	harmonic	up	down	arc	U	complex
Number	3019	3593	1498	2635	5959	1350	1560	274	2076
Percent	15.29 (±1.53)	18.35 (±1.21)	5.26 (±1.24)	12.03 (±1.72)	23.81 (±2.05)	6.88 (±0.80)	8.11 (±0.78)	0.96 (±0.17)	9.32 (±10.81)

**Table 4:** Syllable parameters (means ± SE) from 20985 syllables.

	Number	Duration (ms)	Dominant Frequency (kHz)	Bandwidth (kHz)
all syllables	20985	27.5	74.6618	14.6873
short	2983	5.8 (±0)	77.1717 (±0.1724)	3.5447 (±0.0920)
flat	3446	19.0 (±2)	72.5386 (±0.2120)	3.2346 (±0.0315)
jump	1408	33.7 (±3)	75.5339 (±0.1989)	25.2763 (±0.2209)
harmonic	2513	51.3 (±5)	71.4694 (±0.1850)	46.9210 (±0.1510)
up	5556	25.4 (±1)	76.8575 (±0.0874)	14.2558 (±0.0793)
down	1332	29.2 (±4)	70.5547 (±0.2056)	10.1824 (±0.1028)
arc	1491	34.5 (±3)	73.4158 (±0.1590)	9.9658 (±0.1096)
U	257	31.5 (±6)	77.9739 (±0.5270)	11.3218 (±0.3289)
complex	1999	35.4 (±4)	75.2012 (±0.1705)	12.0179 (±0.1755)

Individual males varied significantly in the vocalizations they produced (Table 5). The total number of vocalizations ranged widely among interactions, but was not significantly different across males (Kruskal-Wallis p = 0.095). The variation in percent use of most syllables was significant across individuals: “short,” “flat,” “harmonic,” “jump,” “up,” “down,” “U,” and “complex”; percent use of “arc” was not (Kruskal-Wallis p<0.05 significance level except “arc”; Fig. S1). Duration, dominant frequency, and bandwidth parameters of total USVs varied across males (Kruskal-Wallis p<0.001). Finally, parameters of different syllable types varied across individuals. Dominant frequency and duration varied significantly for every syllable type across individuals (Kruskal-Wallis p<0.001). Bandwidth was significantly different among males for all except “flat” and “short” syllables (Kruskal-Wallis p-values >0.05 significance level for “flat” and “short,” p-values <0.05 significance level for other syllable type bandwidths). Individual identity of males also had an influence on some non-vocal behaviors. Males significantly differed in the amount of ano-genital investigation they performed, as well as in the amount of ano-genital investigation they received from females (Kruskal-Wallis p = 0.004, p = 0.005).

**Table 5:** Syllable parameters (means ± SE) from 9 males.

	Number	Duration (ms)	Dominant Frequency (kHz)	Bandwidth (kHz)
1	216	32.028 (±0.667)	73.2419 (±0.1797)	15.5588 (±0.4441)
2	107.83	48.448 (±1.106)	68.8672 (±0.2527)	22.3969 (±0.7726)
3	116.83	44.343 (±1.124)	74.1800 (±0.3310)	20.2154 (±0.7841)
4	215	25.739 (±0.339)	78.4541 (±0.1805)	12.2036 (±0.2786)
5	233.8	21.686 (±0.481)	73.5800 (±0.1925)	8.59232 (±0.3010)
6	63.25	25.960 (±0.886)	79.3739 (±0.4392)	6.86008 (±0.2600)
7	24.25	18.604 (±1.696)	71.2854 (±1.2506	11.2042 (±1.6871)
8	146.25	21.232 (±0.657	69.0658 (±0.2530)	7.58086 (±0.4505)
9	330.75	38.333 (±0.689)	67.9877 (±0.2364)	21.8323 (±0.5717)

### USVs are Correlated with other Courtship Behaviors

The production of mouse USVs has been associated in previous work with male sexual behavior, such as sniffing and mounting of females [24], [36]. To determine whether behaviors performed during male-female interactions were related to production of particular syllable types, the percent time males spent performing behaviors across 5 minutes of interaction with a familiar female was analyzed. Since USVs were assumed to be from males, trials were averaged per male to avoid pseudo-replication (n = 9). There were no significant correlations for average behavior per trial between any behaviors scored by video and total number of syllables or percent use of any syllable type after FDR correction for multiple comparisons.

**Figure 2:**
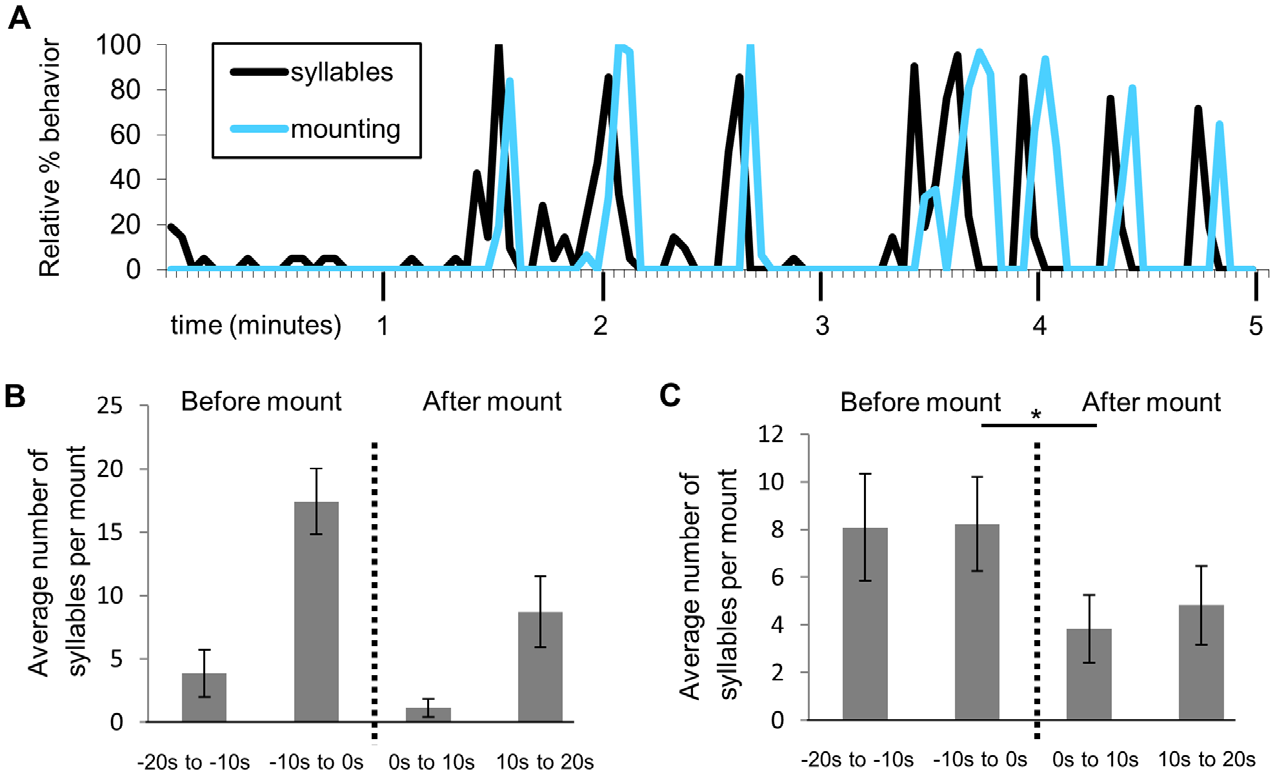
Temporal relationship between USVs and mounting. A) Relative (normalized to max) percent number of all syllables and amount of time spent mounting per 3 second time bin (minor tick marks) for a single 5 minute trial. B) For the same trial, the average number of syllables in the 10 seconds before each mount was higher relative to all other 10 s time bins (bars show SE). C) Across 23 trials with mounting, the number of syllables in the 10 seconds before mounting was significantly higher than the number of syllables in the 10 seconds after mounting (Wilcoxon signed ranks test *p = 0.001); however, there was no difference between the number of syllables in the 10 seconds before mounting and the 20–10 seconds before mounting or the 10 seconds after mounting and the 10 to 20 seconds after mounting.

**Figure 3:**
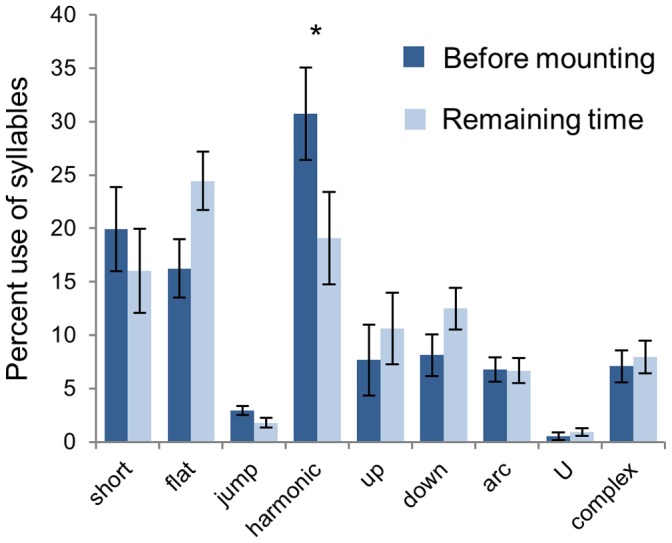
Harmonic syllables increased proportionally before mounting. In 23 trials with mounting, “harmonic” syllables made up an average of 31% (and a total of 50%) of the USVs in the ten seconds before mounting, while “harmonic” syllables only made up an average per mount of 19% (and a total of 17%) of the remaining syllables from the same trials that occurred at times other than 10 seconds before mounting (Wilcoxon signed ranks test *p = 0.023).

A striking temporal relationship between USVs and mounting behavior occurred in some individual trials, with calls increasing in the 10 seconds prior to mounting (Fig. 2A and B). Across trials, the total number of syllables in the 10 seconds before mounting was significantly higher than the number of syllables in the 10 seconds after mounting (Wilcoxon signed ranks p = 0.001; Fig. 2C). However, there was no difference between the number of syllables in the 10 seconds before mounting and the 20–10 seconds before mounting or between the 10 seconds after mounting and the 10 to 20 seconds after mounting (Wilcoxon signed ranks test p = 0.365, 0.392; Fig. 2C), showing a consistent decrease in vocalizations after mounting rather than an increase before. Across syllable types, “harmonic” syllables showed the largest change in use with respect to mounting. During the 10 seconds before mounting, “harmonic” syllables made up an average per mount of 31% (and a sum total of 50%) of the USVs, while “harmonic” syllables only made up an average of 19% per mount (and a sum total of 17%) of the syllables from the remaining time from the same trials (Wilcoxon signed ranks p = 0.023; Fig. 3).

### Female Estrous State Influences USVs

**Figure 4:**
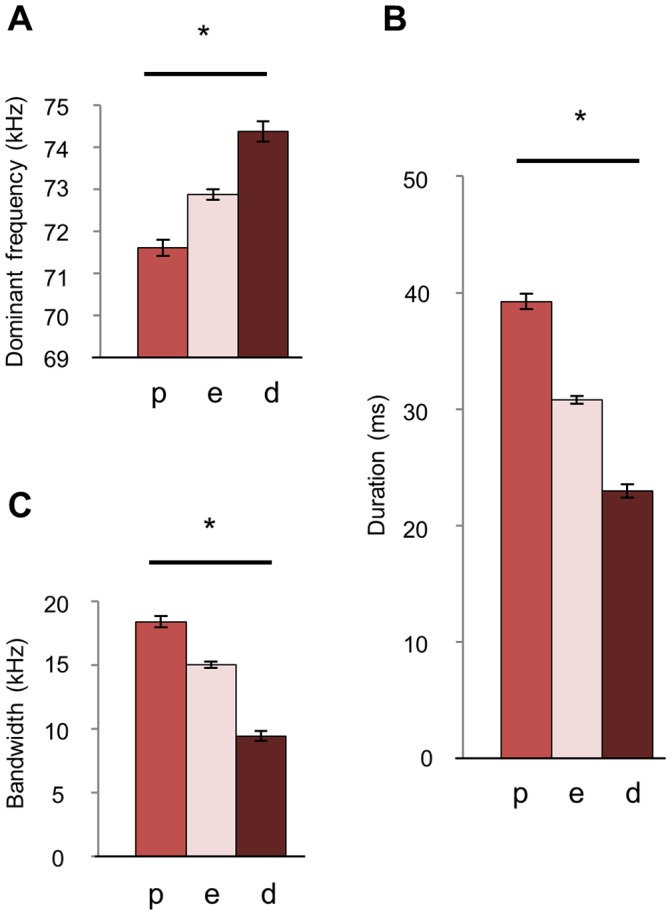
Dominant frequency, duration, and bandwidth of male USVs change with female estrous state. A total of 7149 USV syllables were recorded during 45 male-female interactions. A) The average dominant frequency of USV syllables was lowest during interactions with proestrous females (n = 16 trials), intermediate with estrous females (n = 19 trials), and highest with diestrous females (n = 10 trials). B) Average duration and C) bandwidth were highest with proestrous females, intermediate with estrous females, and lowest with diestrous females (Kruskal-Wallis *p<0.001). Fourteen females were used.

We assessed whether estrous state was associated with male sexual behavior and USVs. Interestingly, males mounted females of all estrous states equally (Kruskal-Wallis p = 0.251). Females of different estrous states received USVs that did not differ in the number of syllables produced nor percent use of different syllables types (Kruskal-Wallis all p-values >0.05 significance level). Syllable parameters, however, varied with respect to female estrous state (Fig. 4). The syllables produced in trials with proestrous females were lowest in dominant frequency and highest in duration and bandwidth, whereas diestrous females received syllables that averaged highest in dominant frequency and lowest in duration and bandwidth (Kruskal-Wallis p<0.001). Average parameters of syllables from trials with estrous phase females were intermediate for all parameters.

Estrous state also influenced most parameters within syllable types. The dominant frequency of each syllable type was influenced by female state such that diestrous females received syllables with a higher average frequency than proestous females (Kruskal-Wallis p<0.05 significance level). For duration, “arc,” “complex,” “down,” “flat,” “harmonic,” and “up” syllables were influenced by estrous state (Kruskal-Wallis p<0.05 significance level). For bandwidth, estrous state influenced “arc,” “harmonic,” “U,” and “up” syllables (Kruskal-Wallis p<0.05 significance level). The differences in duration and bandwidth parameters between estrous state groups for individual syllable types were more complex than the overall effect (Table S2).

### Female Presence Influences USVs

**Figure 5:**
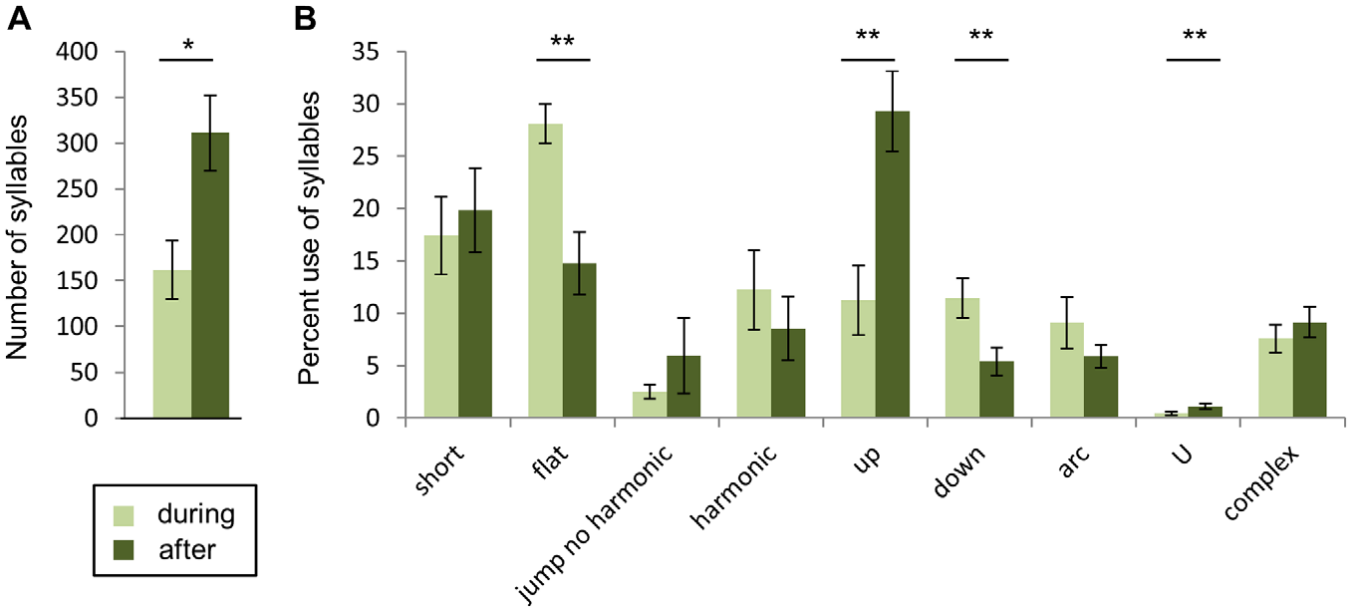
Female removal from male-female interaction changed the number of syllables produced and percent use of syllables types. A total of 21528 syllables were analyzed. A) The number of syllables increased during the 5 minutes after interaction (Wilcoxon signed ranks *p = 0.015). B) The percent use of “up” and “U” syllables increased, while the percent use of “flat” and “down” syllables decreased (Wilcoxon signed ranks test **p = 0.008). For syllables “jump,” “harmonic,” “arc,” “short,” and “complex” there were no significant changes in use between the presence and absence of a female.

By manipulating whether or not a courting male could interact with a female, we analyzed USVs across changes in sub-context within a courtship setting. We compared USVs from a male directly interacting with a female and after the female was removed, leaving the male unable to interact with the female although cues such as scent could have remained. We predicted that USVs would respond to changes in sub-context. After removal of the female from the male’s cage, the average number of syllables increased for the following 5 minutes (Wilcoxon signed ranks p = 0.015; Fig. 5A). The average number of syllables during interaction was 162 (±32), while the average after interaction was 311 (±41). The percent use of “up” and “U” syllables increased, while the percent use of “flat” and “down” syllables decreased (Wilcoxon signed ranks p = 0.008; Fig. 5B). Every male increased in “up” and decreased in “flat” calls across sub-contexts. Every male also increased in use of “U” syllables, but these syllables were rare in both the presence and absence of a female. For other syllables (“jump,” “harmonic,” “arc,” “short,” and “complex” syllables), there was no significant average change in usage from the presence or absence of a female, potentially because the changes in usage of these syllables varied so much among males. Although this variation was observed, for all males, each syllable type was produced during and after interactions with females.

**Figure 6:**
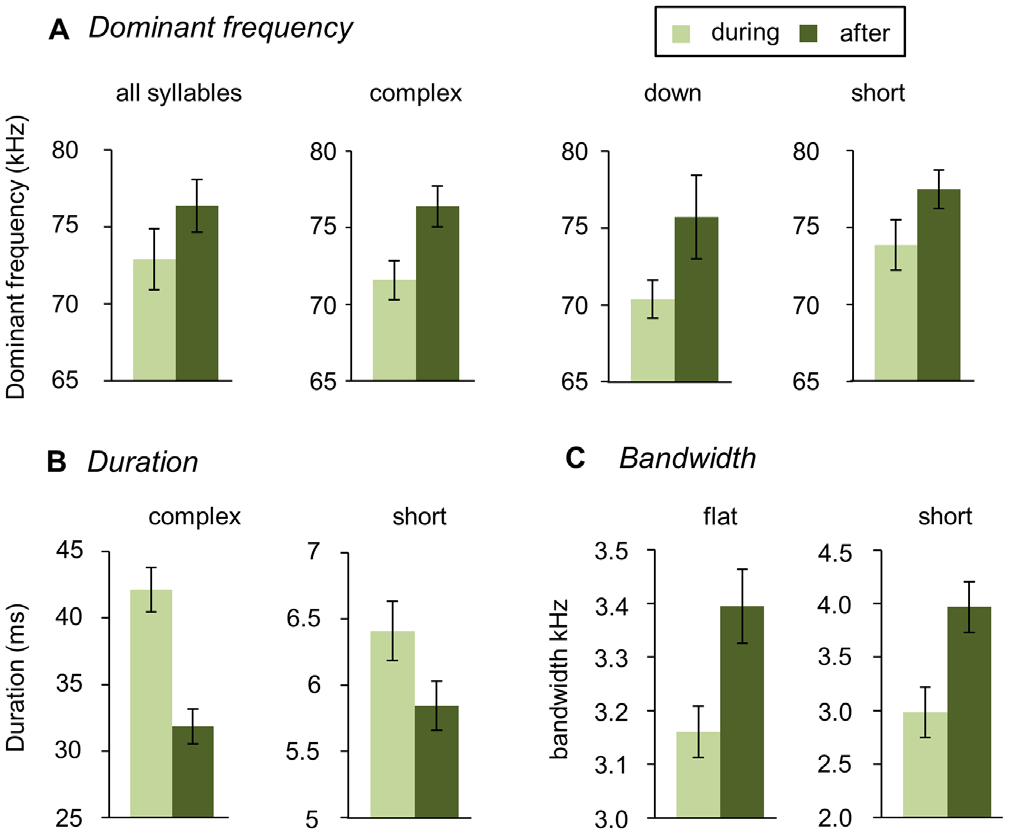
Female removal changed syllable parameters. A) For all syllables combined, the dominant frequency increased after removal of the female, as did the dominant frequency of “complex,” “down,” and “short” syllables. B) Both “complex” and “short” syllables decreased in duration after females were removed. C) Bandwidth increased for “flat” and “short” syllables between sub-contexts. With Wilcoxon signed ranks tests, all comparisons were significant after FDR corrections at the *p<0.05 level.

In addition to syllable usage, the parameters of some syllables changed depending on whether a female was present or absent (Fig. 6). For all syllables combined, the dominant frequency increased after removal of the female (Wilcoxon signed ranks p = 0.008; Fig. 6A). Some parameters of individual syllable types changed as well. Dominant frequency increased for “complex,” “down,” and “short” syllables (Wilcoxon signed ranks p = 0.011, p = 0.021, p = 0.021; Fig. 6A). Both “complex” and “short” syllables decreased in duration after females were removed (Wilcoxon Signed Ranks p = 0.008, 0.015; Fig. 6B). Bandwidth increased for “flat” and “short” syllables between sub-contexts (Wilcoxon signed ranks p = 0.008; Fig. 6C). These changes in parameters were all in the same direction.

Comparing syllable parameters in the presence and absence of females also provided the opportunity to assess whether USVs produced by females as opposed to males substantially contributed to our measurements. If the simple removal of calls produced by females were responsible for the changes in syllable parameters that we observed, then the changes in call parameters should correspond to the identities of female mice, assuming that female USVs vary individually as do those of males. Contrary to this hypothesis, the differences in average duration, dominant frequency, and bandwidth before versus after female removal did not correspond to female identity, but did correspond to male identity (Kruskal-Wallis p = 0.694, 0.588, 0.123 females; p = 0.041, 0.019, 0.013 males).

## Discussion

Mouse USVs are context sensitive, and vary with previous social experience, with development, and across individuals [14], [28]–[30]. This study, to our knowledge, is the first to directly demonstrate that female presence and estrous state influence multiple features of the usage and parameters of many syllables that male mice use in courtship. Furthermore, these changes in syllables occur rapidly in response to short-term changes in salient information within the courtship context. We found support for all three of our initial predictions. That is, syllable production rate and usage correlated with courtship behavior, the estrous state of females influenced USVs, and USVs also changed following female removal. Here, we discuss whether the courtship USVs we measured can best be attributed to males or females, the relationship between USVs and other courtship behaviors, the importance of female presence and estrous state on production of courtship USVs, and the implications of these findings for female responses to courtship USVs.

### Are Changes in USVs Attributable to Males or Females?

Although we presume that the majority of USVs produced during male-female interactions are from male mice, we cannot absolutely exclude the possibility that some USVs were produced by females, because we did not devocalize females in our study. One general interpretation for our results is therefore that the changes we observed in syllable use and parameters were influenced by females producing USVs. Removal of a vocalizing female could change the average frequency of vocalizations and percent use of syllable types recorded, since individuals vary in the parameters of syllables and syllable types used. In addition, since the removed animal was always female and the remaining was always male, this alternate interpretation of our results would suggest sexual dimorphisms in the parameters of USVs in our population of mice. Likewise, an alternative interpretation for the variation we observed across estrous state would be that duration, bandwidth, and dominant frequencies of female USVs change across the estrous cycle.

Despite these important considerations, we think that it is unlikely that female USVs recorded in this study strongly influenced the results for multiple reasons. In previous studies, females produced an insignificant number of USVs in the presence of muted males, and vocalizations were unchanged in the presence of muted females [19], [21]. Another study showed that female mice vocalized when an intruder was introduced, but not when females were intruders, as they were in this study [10]. In our study, no general categories of syllables were observed in the presence of females that were not observed in their absence, suggesting at least that if females produced USVs, these USVs were not grossly different from those of males. Moreover, there were no instances of overlapping USV syllables in our recordings, whereas we have observed overlapping syllables in male-male interactions of this strain (unpublished data). Finally, the differences in call parameters when females were present versus absent did not correspond to female identity, but did correspond to male identity. This finding suggests that calls specific to females did not account for the differences in USV parameters when females were present versus absent, but that changes related to specific males did. On the whole, both previous studies and our own data are therefore most consistent with the hypothesis that changes in call parameters by males underlie the context-dependent differences we observed.

### USVs Correspond to other Courtship Behaviors

Courtship USVs are related to other behaviors exhibited by males during courtship. In the present study, significantly fewer vocalizations were produced in the 10 second window following mounting. This decrease is consistent with the idea that male mice vocalize less during a refractory period after mounting, and supports the general concept that mounting and USV production are related behaviors [24]. In BALB/c mice, particular syllable types are also associated with behavior, such that a long duration (100 ms) syllable only occurs during coitus with proestrous females [36]. Although no syllables this long were observed in CBA/J mice in the current study, “harmonic” type calls were longer in duration than any other type and did increase in use immediately preceding mounting behavior. Correlations between USVs and other courtship behaviors could still be explained by a common cause, such as the level of male arousal. However, in conjunction with studies showing female responses to male USVs, the relationship between USVs and behavior suggests a function for vocal behavior during courtship [23], [26], [27], [43]. For example, longer duration syllables could potentially encourage receptive behavior by females, similar to particular types of song in birds such as canaries and swamp sparrows that can facilitate copulation solicitation displays [44], [45].

One aspect of the USVs produced during courtship that we did not examine was the temporal relationship among syllables. The sequence of syllables produced in bouts has been investigated in CBA/CaJ mice, showing that age-typical sequences occur in non-random order, but the relationship of call sequences to particular behaviors during courtship has not been analyzed [29]. It would be of particular interest to analyze syllable order in the 10 seconds preceding mounting, during which a high number of syllables and the highest proportion of harmonic syllables were produced relative to the time following mounting.

### Females Influence Male Vocal Behavior

Although USVs are a well-described aspect of male mouse courtship behavior, the influence of female presence and estrous state on the production of USVs by males have previously been underappreciated [19], [20], [23], [24], [43], [46] (but see [36], [37]). The results of the present study demonstrate that the structure of USVs is influenced by both female presence and estrous state, each shifting vocal behavior in a different way.

Female presence influenced the percent use of specific syllables, with some increasing and others decreasing in use. Removing females from the arena dramatically increased the number of syllables produced and also increased the dominant frequencies of those syllables. These effects of sub-context have not been reported anywhere else, to our knowledge. One potential function for an increase in call rate following female removal is suggested by experiments that demonstrate female approach to recorded USVs [23], [26]. An increase in USV production by males could potentially encourage such approaches, facilitating female proximity. In addition to the rate of USV production, a set of complex changes occurred in the usage and parameters of some syllables following female removal. One functional hypothesis to explain these changes is that they increase the detectability of the USVs for females at a distance from the males. The increase in bandwidth for “flat” and “short” syllables would support this hypothesis, since a signal of wider bandwidth may be more detectable against background noise [47]. Also supporting an increase in detectability, one of the higher-bandwidth syllables, “up,” increased fourfold in number after female removal. The decreases in the duration of some syllables and the general increase in dominant frequency that we observed would tend to decrease the detectability or propagation of signals, however, as higher frequencies degrade faster in air [48]. A competing functional hypothesis is that females find some signals more attractive. Following female removal, males increased the percent production of “up” and “U” syllables, and decreased the percent production of “flat” and “down” syllables (Figs. 1, 6). Whether females find “up” and “U” syllables more attractive is not known.

Some male birds also show shifts in vocal behavior in response to whether female listeners are present or not. Juncos and song sparrows (and many other passerines) produce vocalizations in specific contexts, usually associated with having a fertile mate nearby, that are distinctly softer and more complex than louder broadcast calls [49], [50]. Less is known about these short-range vocalizations, but they make an interesting comparison to mouse USVs, which are also relatively low in intensity. Male zebra finches also produce two types of songs characterized by whether a female is present or not. The songs differ in parameters including duration, number of motifs, and regularity of syllable sequence, and females prefer aspects of directed song over those of undirected song, although early exposure and endocrine state also influence female preference [51], [52]. In mice, whether shifts in calling by male mice influence female behavior is unknown.

In the present study, female estrous state also influenced USVs. Instead of changing syllable number or usage, estrous state more subtly influenced call parameters. Dominant frequencies of syllables were lowest, durations were longest, and bandwidths were broadest for stimulus females in proestrus. Previous studies have found that rodents change vocalization production depending on female estrous state. Rats vary in the rate of production of courtship calls in response to females depending on estrous state or the level of gonadal hormones in females [53]–[55]. Manipulation of gonadal hormones in female mice affects the ability of chemical signals to evoke male USVs [56]. Some mice also differ in the amount of vocal behavior produced in response to females of different estrous states, but the number of vocalizations produced by male CBA/J mice in the current study did not vary across the estrous cycle of stimulus females [36], [37]. Similar to the changes in the rate of call production close to mounting, one explanation for the changes we did see in parameters of syllables could be that males are more aroused by receptive females and changes in USVs are a by-product of different levels of arousal. A functional alternative is that males modulate their vocalizations based on the possibility for reproductive success. This hypothesis generates the testable prediction that females, particularly those in proestrus, find calls that are longer, lower in frequency, and of greater bandwidth to be most attractive.

### Females Respond to Male Vocal Behavior

Theories on female preference for variation in male vocal behavior have been tested in other models of courtship signaling and reception. In túngara frogs, calls of higher complexity, and lower fundamental frequency are more attractive to females, but also make males more conspicuous to predators [31], [32]. Longer duration and more complex calls are considered to be more energetically expensive in a wide range of animals that use acoustic signals [57]. In many other species, male variation in auditory signaling in these parameters is used by females to select mates, such as in both the Iberian midwife toad and field cricket, in which females respond better to male calls that are more rapid and lower in carrier frequency [33], [34]. Females of some songbird species prefer particular directions of song characteristics, such as fast trill rates [35]. Similarly, in the singing mouse, *Scotinomys teguina,* given the tradeoff between call rate and syllable bandwidth, vocalizations performed near the limit maximizing both parameters are more attractive to females [58]. Investigating the potential costs of producing USVs and female preference in variations of male calls in laboratory mice would increase our understanding of the role of USVs in communication.

Our findings suggest that variation in USVs, including the usage of specific syllables, are part of a suite of directed courtship behaviors. Assessing whether variation in USVs contains communicative significance, however, depends on understanding whether and how females respond behaviorally to such variation. Female mice discriminate among males using olfactory cues, but also demonstrate interest in male USVs and prefer playbacks of vocalizations of unfamiliar individuals over familiar kin [23], [26], [27], [43], [59]. Because individual males vary in vocal behavior (using unique combinations of syllable types and parameters of those syllables), the potential for discrimination between individuals based on auditory information exists [23], [28], [60]. There is also evidence for the genetic basis of variation in USVs, which means that female preference in male vocal behavior could influence the vocal behavior of her offspring [61], [62]. While some female behaviors were measured in the present study, we did not find any correlations between female behavior and USV parameters. However, our experiments were designed to manipulate information relevant to males and not specifically designed to measure female preference for male USV parameters. Testing the relative attractivity of vocalizations with controlled manipulation of frequency and duration parameters using paradigms that adequately measure female discrimination and preference, including post-copulatory mechanisms, would be extremely useful in establishing the significance of the USV variation we have observed.

Female preferences are influenced by sensory biases in processing of signals. Evolution of male courtship signals and matching sensitivity of auditory responses in females are found in a variety of species [63]–[65]. In addition to testing behavioral preferences, measuring neural responses to USVs would improve our understanding of how these signals are received [66]. In other species, sensitivity of female auditory systems varies with seasonal receptivity [67]–[70]. Although social vocalizations have been used to study auditory processing in some mammals, including mice, the influence of female estrous state on processing of courtship vocalizations has not yet been addressed [8], [71], [72].

### Summary

We have found that male mice change the characteristics of the USVs that they produce during courtship in response to changes in relevant social information. This emphasizes the view of such signals as potentially containing context-sensitive information arising from behavioral interactions between signaler and receiver. Our results fit with other studies showing that USVs vary across individuals, across development, and with social experience and suggest that male USVs could carry information about individual identity, age, or behavioral state of the males producing them [14], [28]–[30]. Considerable variation in USVs across species and strain highlights the need to characterize USVs from particular strains of interest [62], [73]. We have begun to characterize the USVs of CBA/J mice because this strain maintains good hearing well into adulthood, an important factor during auditory communication [74]. Our findings increase the usefulness of mouse USVs for understanding context-dependence of both signal production and auditory processing, and consequently for the study of communication disorders.

## Supporting Information

Figure S1 **Males varied in percent use of syllable types.** The percent use of each syllable is shown per male. Black horizontal bars represent means. The variation in percent use of some syllables was significant across individuals: “short,” “flat,” “harmonic,” “jump,” “up,” “down,” “U,” and “complex” (Kruskal-Wallis *p<0.05 significance level except “arc”). The percent use of “arc” syllables was not significantly different across males.
(TIF)

Table S1 Spearman’s correlation coefficient and p-values for percent use of syllable types, total number of syllables, and parameters of syllables were compared with age of males.*
(DOC)

Table S2 Means of syllable type parameters received by females of different estrous states.
(DOC)
