# Supplementary figures and images for: Female Presence and Estrous State Influence Mouse Ultrasonic Courtship Vocalizations
Source: PLoS One. 2012 Jul 18;7(7):e40782. doi: 10.1371/journal.pone.0040782 (PMC3399843; doi:10.1371/journal.pone.0040782)

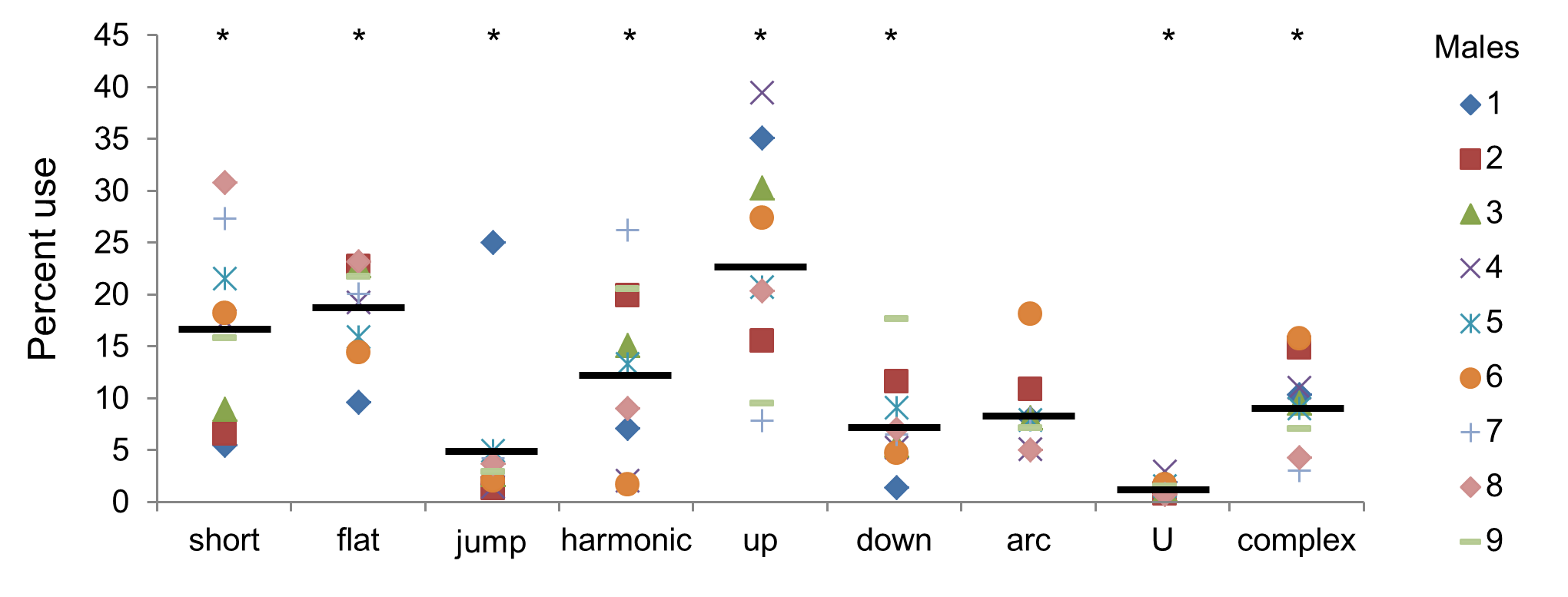

Supplement: Figure S1 — Males varied in percent use of syllable types. The percent use of each syllable is shown per male. Black horizontal bars represent means. The variation in percent use of some syllables was significant across individuals: “short,” “flat,” “harmonic,” “jump,” “up,” “down,” “U,” and “complex” (Kruskal-Wallis *p<0.05 significance level except “arc”). The percent use of “arc” syllables was not significantly different across males. (TIF) [file pone.0040782.s001.tif]
