# Supplementary material for: Female Presence and Estrous State Influence Mouse Ultrasonic Courtship Vocalizations
Source: PLoS One. 2012 Jul 18;7(7):e40782. doi: 10.1371/journal.pone.0040782 (PMC3399843; doi:10.1371/journal.pone.0040782)
Supplement: Table S1 — Spearman’s correlation coefficient and p-values for percent use of syllable types, total number of syllables, and parameters of syllables were compared with age of males.* (DOC) [file pone.0040782.s002.doc]

| Table S1 Spearman’s correlation coefficient and p-values for percent use of syllable types, total number of syllables, and parameters of syllables were compared with age of males.* | | |
| --- | --- | --- |
| Vocal behavior | p-value | Spearman’s coefficient |
| “short” | 0.034 | -0.706 |
| “flat” | 0.847 | -0.076 |
| “jump” | 0.364 | -0.345 |
| “harmonic” | 0.881 | 0.059 |
| “up” | 0.351 | 0.353 |
| “down” | 0.442 | -0.294 |
| “arc” | 0.121 | 0.555 |
| “U” | 0.504 | 0.257 |
| “complex” | 0.023 | 0.740 |
| all syllables | 0.484 | 0.269 |
| duration | 0.150 | 0.521 |
| dominant frequency | 0.682 | 0.160 |
| bandwidth | 0.339 | 0.361 |

*Significance was determined using FDR corrections with a lower *cut off for significance at p = 0.004.*
