# Supplementary material for: Female Presence and Estrous State Influence Mouse Ultrasonic Courtship Vocalizations
Source: PLoS One. 2012 Jul 18;7(7):e40782. doi: 10.1371/journal.pone.0040782 (PMC3399843; doi:10.1371/journal.pone.0040782)
Supplement: Table S2 — Means of syllable type parameters received by females of different estrous states. (DOC) [file pone.0040782.s003.doc]

| Table S2 Means of syllable type parameters received by females of different estrous states. | | | | | | | | | | |
| --- | --- | --- | --- | --- | --- | --- | --- | --- | --- | --- |
| parameter | phase | arc*†‡ | complex*† | down*† | flat*† | harmonic*†‡ | jump† | short† | U†‡ | up*†‡ |
| duration (ms) | proestrous | 39.8 (±1) | 45.5 (±1.8) | 30.0 (±0.9) | 20.0 (±0.4) | 69.1 (±0.9) | 42.1 (±2.8) | 6.0 (±0.1) | 25.6 (±2.1) | 23.0 (±0.6) |
|  | estrous | 37.1 (±0.8) | 37.9 (±0.8) | 33.9 (±0.9) | 20.3 (±0.3) | 61.8 (±1) | 39.8 (±1.8) | 6.1 (±0.1) | 33.7 (±1.4) | 25.9 (±0.3) |
|  | diestrous | 34.1 (±1.8) | 39.1 (±2.4) | 29.0 (±1.6) | 17.6 (±0.4) | 52.1 (±2.9) | 31.5 (±2.6) | 6.0 (±0.1) | 28.0 (±3) | 33.0 (±1.9) |
| dominant frequency (kHz) | proestrous | 73.196 (±0.396) | 70.499 (±0.431) | 68.210 (±0.351) | 69.747 (±0.286) | 70.562 (±0.383) | 70.245 (±1.048) | 74.100 (±0.659) | 68.367 (±1.207) | 74.510 (±0.466) |
|  | estrous | 73.581 (±0.357) | 74.838 (±0.348) | 69.231 (±0.402) | 71.218 (±0.237) | 69.304 (±0.453) | 75.715 (±0.877) | 74.716 (±0.372) | 81.035 (±0.996) | 77.217 (±0.198) |
|  | diestrous | 77.834 (±0.819) | 74.895 (±0.839) | 69.663 (±0.495) | 73.367 (±0.453) | 73.300 (±1.308) | 78.581 (±1.960) | 75.118 (±0.486) | 79.025 (±9.243) | 77.339 (±0.531) |
| bandwidth (kHz) | proestrous | 10.782 (±0.309) | 11.071 (±0.527) | 9.539 (±0.217) | 3.212 (±0.051) | 47.725 (±0.280) | 25.377 (±1.289) | 2.526 (±0.153) | 10.033 (±2.808) | 9.647 (±0.227) |
|  | estrous | 9.898 (±0.232) | 11.930 (±0.332) | 10.000 (±0.205) | 3.104 (±0.042) | 45.975 (±0.366) | 26.176 (±1.144) | 2.995 (±0.183) | 11.544 (±0.526) | 14.649 (±0.209) |
|  | diestrous | 8.839 (±0.527) | 11.300 (±0.547) | 10.229 (±0.332) | 3.082 (±0.068) | 45.455 (±1.356) | 26.915 (±2.303) | 3.034 (±0.292) | 7.525 (±1.103) | 16.348 (±1.212) |

* duration significant across estrous state

†frequency significant across estrous state

‡bandwidth significant across estrous state
